# Bridging the gap: manager support for workers with chronic pain

**DOI:** 10.1093/occmed/kqaf111

**Published:** 2025-11-24

**Authors:** Angela Thornton, Wendy J Chaplin, Holly Blake

**Affiliations:** School of Health Sciences, University of Nottingham, Nottingham, NG7 2HA, UK; School of Health Sciences, University of Nottingham, Nottingham, NG7 2HA, UK; NIHR Nottingham Biomedical Research Centre, Nottingham University Hospitals NHS Trust, Nottingham, NG7 2UH, UK; School of Health Sciences, University of Nottingham, Nottingham, NG7 2HA, UK; NIHR Nottingham Biomedical Research Centre, Nottingham University Hospitals NHS Trust, Nottingham, NG7 2UH, UK

## Abstract

Chronic or persistent pain - such as back pain, headache and arthritis - affects a significant portion of the UK workforce and can limit their ability to work effectively. Line managers are often the first point of employee contact for support and act as a gatekeeper for support services such as occupational health. This perspective highlights the urgent need for tools to empower line managers to consistently support employees with chronic pain and suggests a digital solution based on the Pain-at-Work Toolkit.

## Teaser

Chronic or persistent pain–such as back pain, headache and arthritis –affects a significant portion of the UK workforce and can limit their ability to work effectively. Line managers are often the first point of employee contact for support and a gatekeeper for support services such as occupational health. This perspective highlights the urgent need for tools to empower line managers to consistently support employees with chronic pain and suggests a digital solution.

Key learning points **What is already known about this subject:**

- Approximately 27% of the UK workforce lives with chronic pain. People living with chronic pain report significant impacts on their mental and physical health, and chronic pain also affects people’s productivity at work and their ability to remain working.
- However, many organisations do not have disability workplace policies and practices in place, and therefore not all employers routinely provide support for workers with chronic conditions, including chronic pain.
- Line managers have been identified as highly influential as both the first point of contact when workplace adjustments are required and as a gatekeeper for access to support services such as occupational health.

**What this commentary adds:**

- This commentary highlights the extent to which chronic pain at work is a hidden disability and the lack of consistent, organisational support which is tailored to the employee’s individual needs.
- We provide insight into the knowledge and skills gaps that limit managers’ ability to respond effectively to the fluctuating and individualised nature of chronic pain.
- We highlight the challenges that employers face when looking to accommodate the needs of employees with chronic conditions, and chronic pain in particular; our commentary identifies gaps in organisational provisions and flags a need for additional tools and resources to educate and upskill line managers.

**What impact this may have on practice or policy:**

- One potential solution is the creation of resources for line managers which are aligned with the Pain-at-Work Toolkit; this digital toolkit offers resources to empower employees in managing their chronic pain, building their knowledge and confidence to navigate employee rights and access reasonable adjustments and support at work.
- In recognition of the challenges identified, resources specifically for line managers are currently being developed by the Pain-at-Work team.
- A dyadic approach where both employer and employee have access to complementary resources may benefit both parties; a better understanding of chronic pain at work might enable more proactive identification and implementation of support needs which will improve outcomes for employees with chronic pain.

In the United Kingdom, a systematic review and meta-analysis found that chronic pain affects 35% to 51% of the population, with a pooled estimate of 44% [1]. This equates to around 28 million adults, a figure predicted to increase, largely due to an ageing population [1]. Chronic pain is associated with a high burden to individuals, healthcare services and employers. It significantly impacts on physical and psychological health [2] and can create barriers to gaining and retaining employment [3]. Chronic pain is associated with higher sickness absence and presenteeism (working when unwell) and lower work productivity [2, 4]. One study estimated reductions in work productivity at 2.4 hours (±5.6) per week for adults with chronic joint pain to 9.8 hours (±11.1) per week for adults with multisite chronic pain [2].

However, in practice, organisational policies and practices are variable and employers do not routinely provide support for and advice to employees with chronic pain conditions [2, 5]. Efforts are needed to help employees better manage chronic pain at work and enjoy a better quality of working life. As identified in previous research, line managers play a crucial role in supporting the health and well-being of employees [2, 6]. Line managers are typically the first point of contact when staff seek workplace adjustments [3] but there may be a disconnect between organisational policies and the support line managers provide day-to-day [4]. Employees have stated that line managers’ awareness, understanding and attitude towards chronic conditions such as pain varies considerably, and managers who have experienced a chronic condition themselves are often more willing to assist with access to support than those who have had no lived experience [5]. Our public involvement activities with people who have lived experience of chronic pain [7] [see additional file 4] further highlights the challenges that employees experience in communicating with their line managers about their chronic pain and its impacts on work. The line manager’s attitude can influence every aspect of the process from the employee’s disclosure of disability, to accessing reasonable adjustments and on-going support, and they are a critical gatekeeper and facilitator of support in the workplace. Depending on their understanding and empathy for the impact of chronic pain on work, line managers can either help or hinder access to supportive services such as occupational health; described by employees as a ‘line manager lottery’ [5].

A mixed methods study using an online survey and qualitative interviews further examined employers’ views towards chronic pain and its impacts in the workplace, workplace support for people with chronic pain, including policies and practices, and barriers to implementing supportive measures [8]. Findings confirmed that organisations tend to be ‘reactive’ rather than ‘proactive’ in dealing with chronic conditions in their workforce. There were differences in the level and type of support provided between organisations of different sizes and types. Line managers’ understanding of how to manage an employee with chronic pain was found to be limited and inconsistent, and there was a general lack of clarity about employer responsibilities relating to employees with health conditions [8]. There is a clear need for accessible guidance and tools tailored to help managers support staff dealing with chronic pain in diverse work environments and to support managers in providing advice which consistently reflects organisational values yet makes reasonable accommodations that are tailored to the individual employee. That is not to understate the challenge for employers since chronic pain is a complex, fluctuating entity, and in addition to individual differences in how it manifests, an individual employee’s needs may vary day-to-day.

Although studies [4, 5] identify the need for line manager training or resources, there is currently no research delivering or evaluating the impact of guidelines to help managers navigate the complex needs of employees with chronic pain. Guidance on managing employees with chronic pain is limited to that provided by professional bodies (e.g. Advisory, Conciliation and Arbitration Service (ACAS), Chartered Institute of Personnel and Development [CIPD]) or on various organisational websites (e.g. Business Disability Forum, Personnel Today, and the Reward and Employee Benefits Association), but line managers may be unaware of this guidance or apply it.

Digital interventions can provide cost-effective, scalable, accessible platforms for training and support [9], but a recent systematic review found no studies with evidence-based digital interventions that focused on work-related self-management of chronic pain [10]. To address this gap in employer provisions, the Pain-at-Work Toolkit [2, 11] was co-created with diverse stakeholders, including people living with chronic pain, healthcare professionals and employers. This digital toolkit offers resources to empower employees in managing their chronic pain, building their knowledge and confidence to navigate employee rights and access reasonable adjustments and support in the workplace. However, in recognition that such negotiations can also be ­complex and challenging for managers, resources specifically for ­managers are warranted - currently being developed by the Pain-at-Work team. Consultation with managers suggests that content of manager-targeted resources could include a definition of chronic pain, the impact of living and working with chronic pain, legal responsibilities in supporting people with disabilities, creating psychologically safe work environments to encourage disability disclosure, examples of reasonable workplace adjustments and how to structure a constructive and supportive dialogue with employees.

Since occupational health is typically accessed by referral from line managers, an individual’s line manager has considerable influence on their access to support. A greater understanding of chronic pain and hence more proactive identification and referral to occupational health might improve the speed and ease with which an employee could access this specialist support. For occupational health practitioners this might facilitate more timely interventions and lead to better outcomes for employees with chronic pain.
